# Antimicrobial resistance acquisition after international travel in U.S. travelers

**DOI:** 10.1186/s40794-016-0020-2

**Published:** 2016-03-14

**Authors:** Dana M. Blyth, Katrin Mende, Ashley M. Maranich, Miriam L. Beckius, Kristie A. Harnisch, Crystal A. Rosemann, Wendy C. Zera, Clinton K. Murray, Kevin S. Akers

**Affiliations:** 1 Infectious Disease Service, San Antonio Military Medical Center, JBSA Fort Sam Houston, 3551 Roger Brooke Drive, Houston, Texas 78234-4505 USA; 2 Infectious Disease Clinical Research Program, Uniformed Services University of the Health Sciences, Bethesda, Maryland USA; 3 The Henry M. Jackson Foundation for the Advancement of Military Medicine, Inc., Bethesda, Maryland USA; 4 Pediatric Infectious Disease Service, San Antonio Military Medical Center, JBSA Fort Sam Houston, Houston, Texas USA; 5 U.S. Army Institute for Surgical Research, JBSA Fort Sam Houston, Houston, Texas USA

**Keywords:** ESBL-producing *Enterobacteriaceae*, *Eschericia coli*, Methicillin-resistant *Staphylococcus aureus* (MRSA), Travel

## Abstract

**Background:**

Prior studies have shown an increase in multidrug-resistant (MDR) *E. coli* colonization from two percent in U.S.-based to 11 % in deployed, healthy military personnel. It is unclear if colonization with MDR organisms occurs through deployment exposures or risks related to routine overseas travel. This study prospectively evaluates rates and risk factors associated with MDR gram-negative bacterial and methicillin-resistant *S. aureus* (MRSA) colonization after international travel.

**Methods:**

Participants traveled internationally for five or more days. Pre- and post-travel, colonizing bacteria from oropharyngeal, nares, groin, and peri-rectal (PR) areas were collected using BD CultureSwab™ MaxV(+). Identification and susceptibilities were done utilizing the BD Phoenix™ Automated Microbiology System. Non-MDR pre- and post-travel MDR bacteria within a subject were compared by pulsed-field gel electrophoresis (PFGE). A questionnaire solicited demographics and potential risk factors for MDR acquisition.

**Results:**

Of 58 participants, 41 % were male and median age was 64 years. Pre- and post-travel swabs were obtained a median of ten and seven days before and after travel, respectively. Itineraries included 18 participants traveling to the Caribbean and Central America, 17 to Asia, 16 to Africa, 5 to Europe, 4 to South and North America. Seventeen of 22 travelers used atovaquone/proguanil for malaria prophylaxis. The only MDR organism isolated was extended-spectrum β-lactamase (ESBL)-producing *E. coli* in five (9 %) participants post-travel (all PR and unrelated by PFGE). There were no statistically significant associations between exposure risks and new ESBL-producing *E.coli* colonization. Of 36 participants colonized with *E. coli* pre- and post-travel, new resistance was detected: TMP/SMX in 42 % of isolates (*p* < 0.01), tetracycline in 44 % (*p* < 0.01), and ampicillin-sulbactam in 33 % (*p* = 0.09). No participants were colonized with MRSA pre- or post-travel.

**Conclusion:**

Consistent with prior studies, new antimicrobial resistance was noted in colonizing *E. coli* after international travel. Nine percent of participants acquired new strains of ESBL-producing *E.coli* without identified risks.

## Background

Foreign travel has been increasingly recognized as a contributor to the global spread of extended-spectrum β-lactamase (ESBL)-producing *Enterobacteriaceae* [1–8]. The specific exposures during international travel that place people at risk for acquisition of multidrug-resistant (MDR) bacterial colonization are slowly being identified [7–9]. This increase in ESBL-producing *Enterobacteriaceae* (particularly *E. coli*) colonization has also been identified in healthy, deployed personnel during operations in Afghanistan [10]. Whether the risks associated with these increased rates are related to those of all international travelers or to risk factors particular to the deployed population remains to be answered. Outside the first 24 hours following injury, infections remain a leading cause of morbidity and mortality in military combat casualties so pre-injury MDR bacterial colonization is of particular concern for deployed personnel [11–13]. As such, we evaluated the rates of MDR organism pre- and post-travel colonization within a population of international travelers based out of San Antonio Military Medical Center (SAMMC). The objective of this pilot study was to prospectively assess antimicrobial resistance patterns and associated risk factors in bacterial colonization before and after international travel in a U.S. cohort.

## Methods

### Study design and definitions

Study participants included active duty personnel or Department of Defense beneficiaries ≥18 years of age traveling internationally for five or more days and had been seen for a pre-travel visit at SAMMC between February 1 and November 1, 2013. Exclusion criteria included active infection at the time of enrollment (as determined by the clinical provider), inability to complete the culture swabs or questionnaire, or inability to attend a post-travel visit. At pre- and post-travel visits, subjects completed a questionnaire assessing demographics and exposures potentially related to antimicrobial resistance acquisition during travel including purpose, itinerary, accommodations, water exposure, antimicrobial exposures, hospitalizations, and illnesses. The study was reviewed and conducted as per ethical standards of the Institutional Review Board of the Brooke Army Medical Center.

### Bacterial susceptibility

At the time of enrollment and within 6 weeks of return from travel, patients had culture swabs obtained from the anterior nares, oropharynx, groin, and perirectal area to determine bacterial colonization. BD CultureSwab™ MaxV(+) (Becton, Dickinson and Company, Franklin Lakes, NJ) was used to sample all sites for detection of bacteria. Swabs were plated onto sheep blood, MacConkey agar, and CHROMagar™ *Staph aureus* in order to isolate all gram-negative bacterial colonies and *S. aureus*. Colonies demonstrating morphology consistent with gram-negative or *S. aureus* were subcultured onto sheep blood agar in order to assure culture purity. All isolates were frozen at −80 °C in Trypticase Soy Broth with 15 % glycerol. Individual colonies underwent further automated testing (BD Phoenix™ Automated Microbiology System-Becton Dickinson, Franklin Lakes, NJ) for identification and antimicrobial susceptibility testing.

Non-ESBL pre- and ESBL-producing post-travel *E. coli* within a subject were compared by pulsed-field gel electrophoresis (PFGE) to determine genetic relatedness. The U.S. Food and Drug Administration protocol for gram-negative rods and *Xba* I endonuclease were utilized to evaluate genotypic patterns among the isolates and subsequently group them into pulsed-field types (PFTs). Clonality was assessed using the commercial software BioNumerics (Applied Maths, Inc., Austin, TX) and defined by 85 % similarity. Multiplex PCRs were performed to determine their phylogenetic groups (A, B1, B2, C, D, E, F, Clade I) per the revised Clermont method [14].

### Statistical analysis

The McNemar test was used to compare overall rates of pre- and post-travel ESBL and resistant gram-negative bacteria, *S. aureus*, and resistance to select antibacterial agents. Risk factor analysis was evaluated with *χ* ^2^ and Fisher’s exact test (when expected cell counts were less than 5) for categorical variables and Mann–Whitney U for continuous variables. A *p* value of <0.05 was used as a significant cutoff. Statistical analyses were performed using SPSS software (IBM® SPSS® Statistics Version 19, Chicago, Illinois).

## Results

### Demographics

Of 58 participants, the majority were female and median age was 64 (Table 1). Pre- and post-travel swabs were obtained a median of ten and seven days before and after travel, respectively. The primary regions of travel were Mexico, the Caribbean, and Central America; Asia; and Africa with a median duration of travel of 12 days. The most frequent purpose of travel was vacation followed by visiting friends and relatives.

**Table 1:** Overall demographics and exposures during international travel, number (%) of subjects (*N* = 58)

Male gender	24 (41)
Age, median (minimum-maximum)	64 (15–82)
Region^a^	
Mexico, Caribbean, & Central America	18 (31)
Asia	17 (29)
Africa	16 (28)
Europe	5 (9)
South America	2 (3)
North America	2 (3)
Purpose of travel^a^	
Vacation	43 (74)
Visiting friends and relatives	10 (17)
Other (missionary/volunteer)	5 (9)
Deployment and military duty	4 (7)
School	1 (2)
Duration of travel, median (minimum-maximum)	12 days (6–105)
Living conditions^a^	
Hotel	36 (62)
Friends and relatives	13 (22)
Group living^b^	10 (17)
Boat/cruise	7 (12)
Local water ingestion during travel	27 (47)
Water exposures during travel	20 (34)
Antimicrobial exposure since enrollment	23 (40)
Malaria chemoprophylaxis	22 (38)
Atovaquone/Proguanil	17 (29)
Doxycycline	3 (5)
Chloroquine	2 (3)
Antibiotics for traveler’s diarrhea since enrollment	3 (5)
Ciprofloxacin	2 (3)
Erythromycin	1 (2)
Systemic antibiotics for other indications since enrollment	3 (5)
Azithromycin	1 (2)
Cephalexin	1 (2)
Unknown antibiotic	1 (2)
Illness since enrollment	13 (22)
Duration of illness, median (minimum-maximum)	4 days (1–27)

^a^Percentages greater than 100 as someone can be counted more than once based upon region of travel or living conditions

^b^Group living included barracks, dorms, or kibbutz

### *E. coli* colonization pre- and post-travel

Fifty-two of 58 subjects had *E. coli* identified from either pre- or post-travel swabs and 36 had *E. coli* identified from both pre- and post-travel swabs (serial isolation) (Table 2). Multiple other notable gram-negative organisms were also isolated during screening, but none were associated with MDR or ESBL-production.

**Table 2:** Bacterial colonization according to subjects and locations (*N* = 58)

Organism	No. of subjects	No. of isolates	Anatomic site of isolate recovery			
			Nares	Oropharynx	Groin	Perirectal
*Escherichia coli*	52	105	2	3	13	87
*Staphylococcus aureus*	20	50	23	19	5	3
*Klebsiella* species	21	22	0	6	1	15
*Enterobacter* species	9	12	2	5	1	4
*Citrobacter* species	8	14	5	1	2	6
*Proteus* species	8	13	0	0	2	11
*Morganella morganii*	6	6	0	0	3	3
*Pseudomonas aeruginosa*	5	6	0	3	3	0
*Stenotrophomonas maltophilia*	4	5	0	1	1	3

While no patients were colonized with ESBL-producing *E.coli* pre-travel, five (9 %) participants post-travel were colonized perirectally (*p* = 0.06) (Table 3). The five ESBL-producing *E.coli* represented four different phylo-groups including C, E, F, with two isolates from group A. None of the post-travel ESBL-producing *E.coli* phylo-groups matched those from non-ESBL *E.coli* pre-travel isolates from the same subjects. The ESBL-producing *E.coli* isolates were all unrelated to each other and to the pre-travel isolates from the same subjects by PFGE. There was a trend towards increased rates of ESBL-producing *E.coli* colonization in subjects who described their purpose of travel as missionary/volunteer (*p* = 0.05). While 40 % of those with ESBL-producing *E. coli* colonization post-travel reported an episode of traveler’s diarrhea compared to only 9 % of those without ESBL-producing *E. coli* colonization post-travel, the results did not reach statistical significance (*p* = 0.1). There were no other significant associations between exposure risks and new ESBL-producing *E.coli* colonization after international travel.

**Table 3:** Demographic characteristics of subjects with and without ESBL-producing *E. coli* acquisition during travel, number (%) of subjects (*N* = 58)

	Non-ESBL *E. coli* (*n* = 53)	ESBL-producing *E. coli* (*n* = 5)
Male gender	23 (43)	1 (20)
Age, median (minimum-maximum)	63 (15–82)	67 (58–81)
Region^a^		
Mexico, Caribbean, & Central America	17 (32)	1 (20)
Asia	15 (28)	2 (40)
Africa	15 (28)	1 (20)
Europe	5 (9)	0
South America	1 (2)	1 (20)
North America	2 (4)	0
Purpose of travel^a^		
Vacation	40 (76)	3 (60)
Visiting friends and relatives	10 (19)	0
Other (missionary/volunteer)^*^	3 (6)	2 (40)
Deployment and military duty	4 (8)	0
School	1 (2)	0
Duration of travel, median (minimum-maximum)	12 (6–105)	11 (7–16)
Living conditions^a^		
Hotel	33 (62)	3 (60)
Friends and relatives	12 (23)	1 (20)
Group living^b^	9 (17)	1 (20)
Boat/cruise	7 (13)	0
Local water ingestion during travel	26 (49)	1 (20)
Water exposures during travel	19 (36)	1 (20)
Antibiotic exposure since enrollment	20 (38)	3 (60)
Malaria chemoprophylaxis	19 (36)	3 (60)
Atovaquone/Proguanil	14 (26)	3 (60)
Doxycycline	3 (6)	0
Chloroquine	2 (4)	0
Antibiotics for traveler’s diarrhea since enrollment	2 (4)	1 (20)
Ciprofloxacin	1 (2)	1 (20)
Erythromycin	1 (2)	0
Systemic antibiotics for other indications since enrollment	2 (4)	1 (20)
Azithromycin	1 (2)	0
Cephalexin	1 (2)	0
Unknown antibiotic	0	1 (20)
Illness since enrollment	11 (21)	2 (40)
Duration of illness, median (minimum-maximum)	4 (1–27)	5 (2–8)

^a^Percentages greater than 100 as someone can be counted more than once based upon region of travel or living conditions

^b^Group living included barracks, dorms, or kibbutz

^*^ *p* = 0.05

### Antimicrobial resistance in gram-negative organisms after travel

The ESBL-producing *E.coli* were universally resistant to tetracycline, trimethoprim-sulfamethoxazole (TMP/SMX), and cefazolin. Forty percent were also resistant to ciprofloxacin and gentamicin. Decreased antimicrobial susceptibility post-travel was not unique to ESBL-producing *E.coli*. Of 36 participants colonized with *E. coli* pre- and post-travel, new resistance was detected to TMP/SMX in 42 % of isolates (*p* < 0.01), tetracycline in 44 % (*p* < 0.01), ampicillin-sulbactam in 33 % (*p* = 0.09), cefazolin in 19 %, ciprofloxacin in 17 %, and gentamicin in 14 % of isolates after travel. While new gentamicin resistance was not significantly increased after travel, type of living conditions was associated with new gentamicin resistance (*p* = 0.01). This was primarily related to staying with friends and relatives (VFR) as 80 % of subjects with new gentamicin resistance reported this exposure compared to seven percent without new gentamicin resistance (*p* < 0.01). Similarly, 67 % of subjects with new ciprofloxacin resistance reported VFR during travel compared to seven percent without new ciprofloxacin resistance (*p* < 0.01). There was also a trend toward new TMP/SMX resistance in subjects who reported VFR during travel with 33 % new resistance compared to five percent of those with unchanged TMP/SMX susceptibility (*p* = 0.06). New TMP/SMX resistance was also noted in 47 % of those who reported illness during travel compared to 14 % of those without illness since enrollment (*p* = 0.06).

In contrast to the risk for new acquisition of resistance, several factors associated with decreased rates of post-travel resistance were identified. Thirty-three percent of those without change in TMP/SMX resistance reported staying in group housing (including barracks, dorms, or kibbutz) versus no subjects with new TMP/SMX resistance (*p* < 0.01). Similarly, 35 % of subjects without increased tetracycline resistance reported group housing during travel, compared to none with new resistance (*p* < 0.01). Sixty-five percent of those without increased tetracycline resistance compared to 25 % of those with new tetracycline resistance (*p* = 0.02) and 63 % of those without change in ampicillin-sulbactam susceptibility versus 17 % with new ampicillin-sulbactam resistance reported local water ingestion (*p* = 0.01). Interestingly, all pre- and post-travel *E. coli*, including ESBL-producing *E.coli*, were susceptible to nitrofurantoin.

The remaining gram-negative bacterial isolates were not isolated with enough frequency to perform analysis, but in general, were susceptible to a broad range of antimicrobial agents. Of the subjects with evidence of persistent colonization, one subject had serial isolation of *Klebsiella pneumoniae* without increased resistance. Of the two subjects with serial *Citrobacter* spp. and three with *Proteus* spp. colonization, only one had decreased susceptibility to nitrofurantoin and another to piperacillin-tazobactam post-travel, respectively. No subjects had serial isolation of other gram-negative organisms.

### *S. aureus* bacterial colonization pre- and post-travel

No participants were colonized with MRSA pre- or post-travel (Table 4). Twenty subjects had MSSA colonization during the study, with no significant difference between pre- and post-travel (*p* = 0.51). Of those with MSSA colonization, it was detected on oropharyngeal screening only in 18 % of pre-travel subjects and 36 % of post-travel subjects. No risk factors were identified for change in MSSA colonization (Table 5). MSSA isolates were universally susceptible to doxycycline, minocycline, and rifampin. Only two subjects were colonized with MSSA isolates resistant to clindamycin and TMP/SMX, three to tetracycline, and one to moxifloxacin. No new resistance of colonizing MSSA strains was noted on post-travel screening.

**Table 4:** Subjects with MSSA colonization, number (%) of subjects (*N* = 58)

Site of Colonization	MSSA colonization at any time during study	MSSA colonization pre-travel	MSSA colonization post-travel	McNemar *p* value	Acquisition or Persistence of MSSA colonization between Pre- and Post-travel
Subjects	20 (35)	17 (29)	14 (24)	0.508	6 without persistent isolation, 3 new acquisitions (11 persistent)
Nares	14 (24)	14 (24)	9 (16)	0.06	5 without persistent isolation, 0 new acquisitions (9 persistent)
Oropharynx	14 (24)	9 (16)	10 (17)	1.00	4 without persistent isolation, 5 new acquisitions (5 persistent)
Groin	5 (9)	4 (7)	1 (2)	0.38	4 without persistent isolation, 1 new acquisitions (0 persistent)
Perirectal	3 (5)	2 (3)	1 (2)	1.00	2 without persistent isolation, 1 new acquisitions (0 persistent)

**Table 5:** Demographic characteristics of subjects with and without change in MSSA colonization, number (%) of subjects (*N* = 58) ^c^

	No change in MSSA (*n* = 49)	Change in MSSA (*n* = 9)
Male gender	18 (37)	6 (67)
Age, median (minimum-maximum)	64 (15–82)	55 (18–81)
Region^a^		
Mexico, Caribbean, & Central America	16 (33)	2 (22)
Asia	14 (29)	3 (33)
Africa	14 (29)	2 (22)
Europe	4 (8)	1 (11)
South America	1 (2)	1 (11)
North America	2 (4)	0
Purpose of travel^a^		
Vacation	37 (76)	6 (67)
Visiting friends and relatives	7 (14)	3 (33)
Other (missionary/volunteer)	5 (10)	0
Deployment and military duty	3 (6)	1 (11)
School	1 (2)	0
Duration of travel, median (minimum-maximum)	12 (6–105)	12 (8–42)
Living conditions^a^		
Hotel	31 (63)	5 (56)
Friends and relatives	10 (20)	3 (33)
Group living^b^	10 (20)	0
Boat/cruise	6 (12)	1 (11)
Local water ingestion during travel	24 (49)	3 (33)
Water exposures during travel	18 (37)	2 (22)
Antibiotic exposure since enrollment	18 (37)	5 (56)
Malaria chemoprophylaxis	17 (35)	5 (56)
Atovaquone/Proguanil	15 (31)	2 (22)
Doxycycline	1 (2)	2 (22)
Chloroquine	1 (2)	1 (11)
Antibiotics for traveler’s diarrhea since enrollment	3 (6)	0
Ciprofloxacin	2 (4)	0
Erythromycin	1 (2)	0
Systemic antibiotics for other indications since enrollment	3 (6)	0
Azithromycin	1 (2)	0
Cephalexin	1 (2)	0
Unknown antibiotic	1 (2)	0
Illness since enrollment	13 (27)	0
Duration of illness, median (minimum-maximum)	4 (1–27)	n/a

^a^Percentages greater than 100 as someone can be counted more than once based upon region of travel or living conditions

^b^Group living included barracks, dorms, or kibbutz

^c^No statistically significant differences identified between the two groups

## Discussion

To further elucidate the risk factors associated with pre-injury colonization with MDR gram-negative organisms in our deployed population, we first sought to evaluate the risk factors associated with MDR organism colonization in international travelers. Within this prospective pilot study, we determined that international travel was associated with a trend towards increased rates of ESBL-producing *E.coli* colonization. Because of small numbers, no individual risk factors for ESBL-producing *E.coli* colonization acquisition within travel could be determined. However there were multiple risk factors, as well as protective factors, for acquisition of individual drug resistance within serial *E. coli* isolates.

Prior studies have identified international travel as a risk factor for ESBL-producing *E.coli* colonization, but have largely been performed in cohorts outside of the United States [1, 3, 5, 15–17]. Similar to the only prior prospective study of pre- and post-travel ESBL-producing *Enterobacteriaceae* colonization within an American cohort (based out of New York City), the only ESBL-producing *Enterobacteriaceae* isolated was *E. coli.* Within the New York City based cohort ESBL-producing *E.coli* colonization increased from 2.5 % pre- travel to 25 % post-travel [2]. Similar post-travel rates were noted within European and Canadian travelers [1, 3, 9, 15]. This is in comparison to the markedly lower rates of 0 % pre- and 9 % post-travel ESBL-producing *E.coli* noted within this study. Notably, the European and New York City based cohorts all had a higher proportion of subjects traveling to Asia and Africa, which have been shown to be areas with higher risk for ESBL-producing *Enterobacteriaceae* acquisition [1–3, 15]. Also notable, the study based out of New York City had a longer duration of international travel (median 21 days compared to 12 days within our cohort) [2].

Co-resistance to other antimicrobials is common with ESBL-producing *E.coli*. All ESBL-producing *E.coli* isolates were resistant to tetracycline, TMP/SMX, and cefazolin, higher than rates reported in evaluation of ESBL-producing *E.coli* domestically [18]. Within our post-travel ESBL-producing *E.coli* isolates, similar to prior studies in European travelers, there was 40 % co-resistance to ciprofloxacin and gentamicin [1, 15, 16]. Reassuringly, all *E. coli* isolates remained susceptible to nitrofurantoin as data indicates that fecal flora serve as the initial source of uropathogenic *E. coli* [1, 19].

While international travel has consistently been shown to be associated with antimicrobial resistance acquisition, few specific risk factors associated with international travel have been identified. In fact, no prior studies have examined risk factors for ESBL-producing *E.coli* acquisition particular to travel within an American cohort. Prior studies from European and Canadian cohorts have shown location of travel to be a significant risk factor (including South and East Asia, the Indian subcontinent, Middle East, and Africa) [1, 3, 7, 15, 16]. A recent study also revealed the United States as a risk factor for ESBL-producing *E. coli* colonization after international travel for the first time [20]. This emphasizes the point that local resistance rates play a major role in the attributable risk of resistance acquisition of travelers. Other risk factors identified within specific cohorts include development of gastroenteritis or other gastrointestinal symptoms during travel, traveler’s diarrhea, antimicrobial exposure, and older age [1, 9, 16]. Interestingly, within a Swedish cohort, all three travelers who were treated with ciprofloxacin for gastroenteritis acquired ESBL-producing *E.coli* on post-travel evaluation, compared to one of two subjects in our cohort [1]. As the overall post-travel ESBL-producing *E.coli* rate within our cohort was low, individual risk factors did not reach statistical significance. There was a trend towards those who described their purpose of travel as missionary/volunteer as having a higher rate of ESBL-producing *E.coli* post-travel.

Within this cohort, the ESBL-producing *E.coli* were from four distinct phylo-groups, none of which were identified as the dominant B2 phylo-group which has been associated with the global ST131 clone. A Canadian study of 31 post-travel ESBL-producing *E.coli* isolates revealed 10 were of the B2 phylo-group (8 of which were ST131 clones), 11 phylo-group A, 2 B1, and 8 phylo-group D [3]. Another study of 83 French soldiers following evacuation from overseas identified 11 ESBL-producing *E. coli*, of which eight were from the B2 and D phylo-types and three from the commensal phylo-groups A and B1 [21]. Prior studies have shown that there is a higher rate of persistent colonization, traveler’s diarrhea, and extraintestinal infections with the more virulent B2 and D phylo-groups compared with commensal *E. coli* phylo-groups [14, 17, 19, 22–24]. It should be noted that these studies were not performed with the recently revised Clermont *E. coli* phylo-typing method which was used for this study which identifies eight phylo-groups rather than four (though likely does not affect isolates previously assigned to B1 and B2 phylo-groups) [14]. A recent study evaluating UTI-associated *E. coli* isolates with the revised Clermont method revealed a more heterogeneous sample than previously thought with only 56 % of the isolates belonging to B2 and D phylo-groups and 32 % of the urine isolates from lineages A, B1, and E [25]. The fact that post-travel ESBL-producing *E.coli* isolates were unrelated to pre-travel isolates in the four patients with serial *E. coli* isolation could represent new strain acquisition, or polymicrobial colonization which was not captured by the choice of single isolates for testing (though morphologically distinct appearing colonies were evaluated).

Similar to prior studies, there was a significant decrease in antimicrobial susceptibility of *E. coli* isolates post-travel [26–28]. Among *E. coli* isolates, there was a statistically significant decrease in TMP/SMX and tetracycline susceptibilities, as well as a trend toward decreased ampicillin-sulbactam susceptibility. Prior studies have shown similar rates of TMP/SMX resistance post-travel [26, 27]. Visiting friends and relatives was associated with increased ciprofloxacin and gentamicin resistance during travel. This is of particular concern as ciprofloxacin and gentamicin resistance has been associated with prolonged carriage in prior studies of travelers, with up to ten percent of participants still harboring ciprofloxacin or gentamicin resistance at six months following travel [17].

Interestingly, both group housing and local water ingestion during travel were associated with decreased acquisition of antimicrobial resistance during travel, particularly to ampicillin-sulbactam and tetracycline. Similar findings were noted in a study by Kennedy and Collignon who reported that returning without resistant *E. coli* was statistically associated with consumption of water that was not bottled or boiled [26].

Unlike a prior study of US- and Afghanistan-based military personnel, no MRSA was detected at any point [29]. As in prior studies, recovery of *S. aureus* was increased by extra-nares screening [30, 31]. Eighteen percent of pre-travel and 36 % of post-travel *S. aureus* isolates were recovered from only the oropharynx. Also differing from the prior military population where the US-based personnel had higher rates of oropharyngeal colonization, rates of oropharyngeal colonization increased from 53 % pre-travel to 72 % post-travel within our cohort [29].

Limitations of our study include the small cohort size and subsequent low rate of ESBL-producing *E. coli* colonization. Additionally, there were relatively few travelers to previously identified high risk regions for resistance acquisition. These factors led to decreased power to detect individual risk factors for antimicrobial resistance acquisition. Because the median age of our study population is 64 years, this limits applicability of these findings to our active duty military population, but does make it more generalizable to the average international traveler. Screening for ESBL-producing *E. coli* colonization was performed with perirectal swabs rather than stool culture, the gold standard, which may have decreased yield. However, Lautenbach et al. have shown the sensitivity of perirectal swabs compared to stool samples for identification of resistant gram-negative bacilli to be 90 %. They also showed that perirectal swabs were in complete agreement with rectal swabs and were most likely to be falsely negative when the burden of resistant gram-negatives within stool culture was very low [32]. Use of rectal swabs has been used successfully in similar prior studies [15].

## Conclusion

Overall, our findings support those seen in prior studies, with increased rates of antimicrobial resistance following international travel. Visiting friends and relatives was associated with acquisition of resistance to various antimicrobials including ciprofloxacin, one of the first line antimicrobials for treating *E. coli* infections. While, traveler’s diarrhea has been shown to not only be associated with an average 24-h period of disability, but also long-term complications including persistent or intermittent gastrointestinal symptoms in a minority of travelers (including subsequent diagnosis of irritable bowel syndrome in between five and ten percent), its occurrence as well as treatment has been associated with increased risk of antimicrobial resistance acquisition [1, 9, 16, 33]. Further elucidation of risks for antimicrobial resistance acquisition while traveling will better enable providers to counsel their patients regarding risks of travel. It will also allow providers to further balance the risks and benefits of empiric treatment of traveler’s diarrhea with antimicrobials [34]. Reassuringly, our rates of ESBL-producing *E.coli* acquisition were significantly lower than those seen in other studies of international travelers. Larger, prospective studies are needed to further define individual risk factors particular to travel which are associated with ESBL-producing *Enterobacteriaceae* acquisition.

## Abbreviations

ESBL: extended-spectrum β-lactamase
MDR: multidrug-resistant
MRSA: methicillin-resistant *S. aureus*
PR: peri-rectal
PFGE: pulsed-field gel electrophoresis
SAMMC: San Antonio military medical center
PFTs: pulsed-field types
TMP/SMX: trimethoprim-sulfamethoxazole
VFR: staying with friends and relatives
